# A Sorting Task with Emojis to Understand Children’s Recipe Acceptance

**DOI:** 10.3390/foods14111839

**Published:** 2025-05-22

**Authors:** Olatz Urkiaga, María Mora, Elena Romeo-Arroyo, Sara Pistolese, Angélique Béaino, Giuseppe Grosso, Pablo Busó, Juancho Pons, Laura Vázquez-Araújo

**Affiliations:** 1 GOe Tech Center, Technology Center in Gastronomy, Basque Culinary Center, 20009 Donostia-San Sebastián, Spain; ourkiaga@bculinary.com (O.U.); eromeo@bculinary.com (E.R.-A.); mmora@bculinary.com (M.M.); 2 Basque Culinary Center, Faculty of Gastronomic Sciences, Mondragon Unibertsitatea, 20009 Donostia-San Sebastián, Spain; 3 Provincia d’Italia dei Fratelli Maristi delle Scuole, Via Fratelli Maristi 2, 80014 Naples, Italy; 4 Frères Maristes au Liban, Dik el Mehdi, Beyrouth 70540, Lebanon; 5 Department of Biomedical and Biotechnological Sciences, University of Catania, 95123 Catania, Italy; giuseppe.grosso@unict.it; 6 Center for Human Nutrition and Mediterranean Foods (NUTREA), University of Catania, 95123 Catania, Italy; 7 Technological Institute for Children’s Products & Leisure AIJU, 03440 Alicante, Spain; pablobuso@aiju.es; 8 Editorial Luis Vives (EDELVIVES), Carretera de Madrid, 50012 Zaragoza, Spain

**Keywords:** gastronomy, acceptance, appearance, meals, Mediterranean diet

## Abstract

Food acceptability in children is a complex, multi-dimensional process influenced by sensory perception, expectations, and context. The present study investigated children’s perception and acceptance of 20 Mediterranean recipes chosen from five different gastronomy cultures (Lebanese, Egyptian, Italian, Spanish, and Portuguese) using photographs as stimuli. A total of 184 children (10 to 13 years old) from three countries (Italy, Lebanon, and Spain) participated in a sorting task with emojis to express liking. In addition, Spanish and Lebanese participants completed a Check-All-That-Apply (CATA) activity to label the recipe groups they had created. The results from the sorting task, analyzed using ANOVA, revealed that recipes including meat/poultry and cereals were most preferred, while legume-based and vegetable dishes received lower acceptance. Children grouped recipes primarily by main ingredient, irrespective of the origin of the recipe (gastronomy culture). Spanish children showed higher acceptance of foreign recipes compared to Lebanese and Italian, demonstrating a significant “country x recipe origin” interaction. The CATA analysis revealed that children associated descriptors such as “healthy”, “tasty”, or “delicious” with highly rated recipes and descriptors such as “too many vegetables” and “bad taste” with lower-rated dishes. While participants showed a positive predisposition towards the “healthy” term, a negative response to recipes based on vegetables and legumes was evident.

## 1. Introduction

Food acceptability is a process of a multi-dimensional nature, related to the consumer’s interaction with the food in a specific context, and depending on the consumer’s own expectations, degree of satisfaction, and needs [1]. Children represent a consumer group with unique needs and highly selective food preferences, which are shaped by a combination of internal and external influences such as sensory processing or parental modeling [2]. Guiding children toward healthy food choices requires a clear understanding of the factors influencing their decisions. Gallo et al. [3] found that children primarily emphasized sensory properties (e.g., aroma and texture) when describing their food preferences and dislikes during focus group sessions. Additionally, the results of the qualitative research sessions conducted by these investigators showed that familiarity and expectations were significant when discussing children’s selections of items from a school lunch menu.

Acceptance of school menus and recipes has been researched using different approaches. Santana et al. [4] conducted a systematic review including 89 studies with the different methods used to evaluate the acceptability of school meals offered by school feeding programs around the world. The results showed that the most used methodology for acceptability assessment was the Hedonic Scale/Likert Scale, used in approximately 70% of studies. In addition to the use of hedonic scales, these authors indicated that other kinds of questionnaires/interviews (with objective and subjective questions) were used in 45% of studies included in their review, and the use of mathematical formulae/visual estimation of consumption and waste production appeared in 40% of studies. Analysis of this review indicated that some aspects, such as familiarity [5,6] and the food preparation procedure [7,8], were significant determinants of meal acceptance.

Given the unique characteristics of children as consumers and the inherent challenges of conducting effective tests with this population, such as maintaining engagement and attention, researchers have explored various methodologies to assess children’s food acceptance and preferences. Because of their proven utility in being used by children to discriminate among products and measure the emotions elicited by foods [9], emojis have been extensively used in many studies, trying to collect data on emotions as well as food acceptance in qualitative and quantitative consumer research activities (e.g., [10,11]). For example, Gallo et al. [3] used emojis during different focus group sessions in which children described their feelings about their selected favorite, “just okay”, and least favorite foods before, during, and after recalled consumption moments. Using a completely different approach, Schouteten et al. [10] showed the use of emoji for emotional profiling and choice prediction in a quantitative study (*n*~150) conducted with children who assessed five different samples of a biscuit.

Also, to adapt sensory science methods for children, visual food images or pictures [12,13,14], as well as food models [13], have been used as stimuli instead of current food samples to explore children’s reactions. In general, the results of these studies seemed to indicate that using real foods as the stimuli generated the most reliable data, although photographs could be a convenient alternative with adequate performance, especially when children were over 9 years old. Finally, different kinds of general sensory methods have been proposed and adapted to better understand food preferences and perception of this young consumer segment, as well as to involve them in food design processes, including qualitative co-creation methods [15,16,17] and even rapid sensory methods such as projective mapping with food stickers and word association tasks [18]. While significant progress has been made in developing alternatives to understanding children’s food reactions, research on the cross-cultural assessment of whole meals and children’s responses to different cultural recipes remains limited.

Although allowing children to assess the current meals served in the real context of canteens may be the best option to gather acceptance data, logistical and resource challenges may often restrict their implementation. In addition, conducting studies in several schools or countries could be challenging because of disparities in the availability of equivalent ingredients and kitchen equipment across schools, as well as differences in the culinary skills and personal style of individual cooks, which could impact the final sensory properties of the prepared dishes. Considering the difficulties in gathering data on the cross-cultural perception of dishes and meals, the aim of the present study was to assess the suitability of a rapid method to determine acceptance of a set of recipes from the Mediterranean basin by children from countries of the Mediterranean area. Building on the hypothesis that appearance significantly influences food acceptability, and because food photographs have been proven to provide useful information to determine acceptance by children [13], this study investigates children’s perception of a set of 20 recipes from Spain, Egypt, Italy, Portugal, and Lebanon, using photographs as stimuli and a sorting task with emojis as the main approach.

## 2. Materials and Methods

The present study was conducted within the context of the European-funded DELICIOUS project, whose study protocol has been previously described [19] and approved by the ethics committee of Mondragon University (no. IEB-20230704). Informed consent was obtained from the parents of all children participating in the corresponding project activities described in the protocol.

### 2.1. Participants

A total of 184 children from three schools participated in the present study at Istituto Fratelli Maristi in Giugliano in Campania, Italy (*n* = 50), Collège Mariste Champville in Dik EL Mehdi and Collège Notre Dame de Lourdes des Frères Maristes in Jbail-Amchit, Lebanon (*n* = 72), and Lekeitioko Herri Eskola and the Lekeitioko Azkue Natura Ikastola in the Basque Country, Spain (*n* = 62). The age range was 10–13 years old (mean = 12.0; s.d. = 1.0), and the gender ratio was balanced (49% identified themselves as females and 51% as males). Data were collected during the 2024–2025 school year, from November 2024 to April 2025.

### 2.2. Samples

A total of 20 recipes from 5 different gastronomies (Lebanon, Egypt, Italy, Spain, and Portugal) of the Mediterranean basin were cooked and photographed by professional chefs and a photographer, 4 from each country, and representing a variety in the composition of the dishes but with a main ingredient that was clearly visible in the photos (4 recipes from each of the following groups: vegetables, meat/poultry, cereals, fish, and legumes). Table 1 shows the recipes list including their classification by main ingredients and country of origin. The photos, as well as details about the recipes (ingredients and preparation procedures), can be found in the book Simply DELICIOUS [20].

### 2.3. Procedure: Sorting Task

A sorting task was chosen based on previous reports indicating its effectiveness and ease of comprehension among children [21]. The set of 20 photos, randomized and coded using 3-digit numbers, together with seven emojis (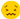, 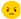, 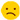, 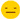, 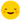, 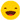, 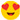; [11]) and seven paper clips were given to each participant inside an envelope. Participants were instructed to label the envelope with their age and gender (no additional information) and (1st) spread the emojis in their table, (2nd) look at the photos one by one, and group them under the emoji they considered better expressed their opinion about the dish presented in the image (not the appearance, but imagining eating the whole dish), and (3rd) clip the corresponding emoji with the photos, put them inside the envelope, and return it to the researcher. Each recipe was assigned to a single emoji, ensuring a one-to-one correspondence between recipes and emotional responses. Because emojis were the grouping factor, participants could create up to seven groups, but using all emojis was optional. Therefore, the number of groups created by each participant was variable.

Additionally, the Spanish and Lebanese groups had 7 sets of 17 descriptive labels (‘too much vegetables’, ‘too little vegetables’, ‘too much fish’, ‘too little fish’, ‘too much meat’, ‘too little meat’, ‘colorful’, ‘colorless’, ‘tasty’, ‘tasteless’, ‘spicy’, ‘familiar’, ‘exotic’, ‘delicious’, ‘healthy’, ‘bad taste’, and ‘odd/weird’) they could add to each “emoji + recipes” group in a “Check All That Apply” task to further characterize each emoji-recipe group. These labels could be used multiple times or not at all and were clipped to the corresponding group before being returned to the researcher. The different concepts of the labels were obtained from qualitative research activities previously conducted with children from the same countries during the development of the DELICIOUS project [17], ensuring the terminology was familiar and understood by them in food contexts [17]. Once selected, the terms were back-translated into the language of each children group (Spanish, Italian, and French).

### 2.4. Data Analysis

The responses from children were analyzed considering 2 different approaches. On the one hand, sorting task data were analyzed using STATIS, as previously reported by other authors [22], to determine the different groups made by children in each country and across both countries, as well as to detect potential behavioral patterns. The data matrix was structured with recipes in rows and children in columns, where each participant assigned each photograph to a group. STATIS was chosen because it accounts for individual differences, weighing each participant’s contribution based on their agreement with the overall consensus. On the other hand, emojis were transformed into the 7 points of a hedonic scale [11] and analyzed using two-way ANOVA in which the recipes, the country (Spain, Italy, and Lebanon), the origin of the recipes (Lebanon, Egypt, Italy, Spain, and Portugal), and the main ingredients (vegetables, meat/poultry, cereals, fish, and legumes) were considered factors. Finally, a correspondence analysis and a Chi-Square test were used to study the relationship among recipes, emojis, and labels using a contingency table built with the Spanish and Lebanese children’s responses. Differences were considered significant when *p* < 0.05 unless otherwise stated. All statistical analyses were performed using XLSTAT (Version, 2024.4.1, Addinsoft, Denver, CO, USA).

## 3. Results

**Figure 1:**
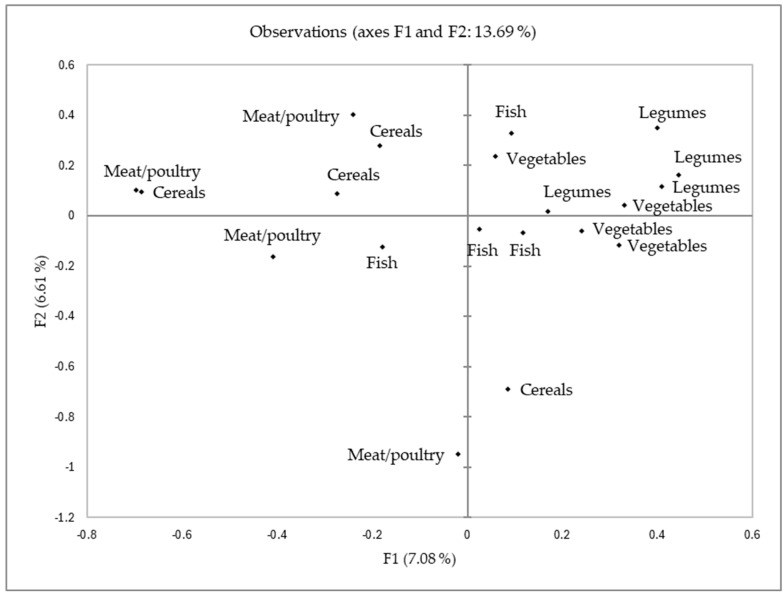
Results of the consensus plot conducted with the samples listed in Table 1.

Figure 1 shows the consensus plot resulting from STATIS analysis of the sorting task, with the relationship among recipes by main ingredient. Although the explanation percentage was quite low (<14%), the distribution presented in Figure 1 suggested that children tended to group the recipes considering their main ingredients. Conducting the same analysis considering other factors such as “recipe origin” did not show a higher explanation or response pattern. The explanation percentage of the model suggested a low agreement on the classification of the recipes, illustrating the heterogeneity of children’s responses. The use of emojis to group recipes forced children to provide subjective evaluations, contributing to detecting the differences in preferences but resulting in a low explanation percentage.

Table 2 shows the general results from the ANOVA test by factor after considering each emoji one of the 7-point hedonic scales reported by Swaney-Stueve et al. [11]. In general, Lebanese and Spanish children liked all recipes more than Italian children. The results suggested that the recipes including meat and poultry were the ones with a higher acceptance, followed by recipes including cereals. The recipes receiving the least acceptance were the ones based on legumes and vegetables. Recipe origin influenced responses, with Lebanese recipes preferred over others and Spanish recipes least liked. Finally, the results showed that the 20 recipes were liked differently, “grilled lamb kabobs (LB)” (mean liking score = 5.3), “whole wheat pasta and chicken salad (IT)” (mean liking score = 4.6), and “beef meatballs with fennel salad (IT)” (mean liking score = 4.5) being the three recipes that received the highest scores, and “monkfish brodetto (IT)” (mean liking score = 3.5), “pisto (SP)” (mean liking score = 3.5), and “kolkas (EG)” (mean liking score = 3.1) being the least liked ones.

**Figure 2:**
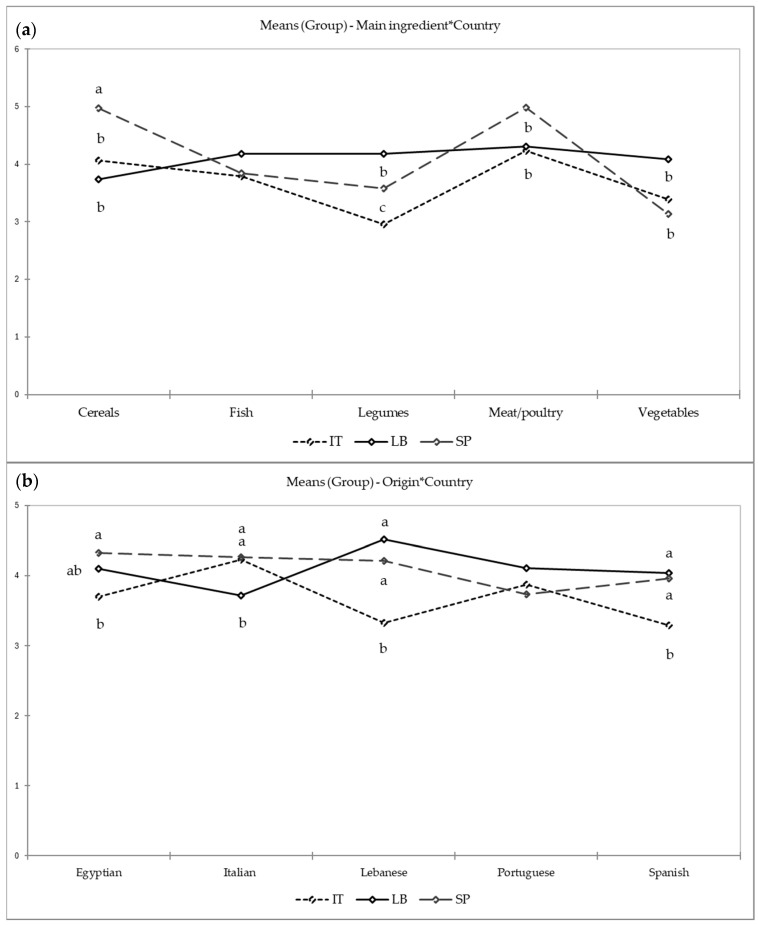
Two-way ANOVA results of the interaction Country × Main ingredient (**a**) and Country × Origin (**b**). Different letters indicate significant differences (*p* < 0.0001) among countries (Tukey’s HSD).

Figure 2 shows the significant differences found in the results of the two-way ANOVA for the interactions “Main Ingredient × Country” and “Origin × Country”. Spanish children liked the recipes based on cereals and meats/poultry more than Italian or Lebanese children, while Lebanese children liked legumes and vegetables more than Italian and Spanish children. A greater preference for legumes was noted among Spanish children compared to Italian children, although the general preference profiles of these groups displayed similarities, contrasting with the distinct preferences of Lebanese children.

Contrary to expected, some foreign recipes were liked over the recipes of their own gastronomic cultures, for example, some Egyptian recipes were liked over recipes from their own countries. A significant interaction between “Origin x Country” was identified, with Spanish and Lebanese children showing higher acceptance of recipes from Egypt, Lebanon, and Spain than Italian children; recipes from Portugal were similarly liked in the three countries. Italian and Lebanese children showed a preference for recipes from their own cultures, with Italian recipes receiving higher punctuations in Italy and Lebanese recipes receiving higher punctuations in Lebanon. Recipes from other countries received significantly lower ratings (*p* < 0.05); in both countries, Portuguese and Egyptian recipes ranked second and third, respectively. On the contrary, Spanish children rated recipes from Egypt, Italy, Lebanon, and Spain as having similar punctuations being Portuguese the ones with the lowest score. Since neither the names nor details about the recipes were provided to the children, they were unaware of the potential origins or ingredients of the dishes; therefore, their responses were based solely on the appearance and identifiable ingredients in the photos.

**Figure 3:**
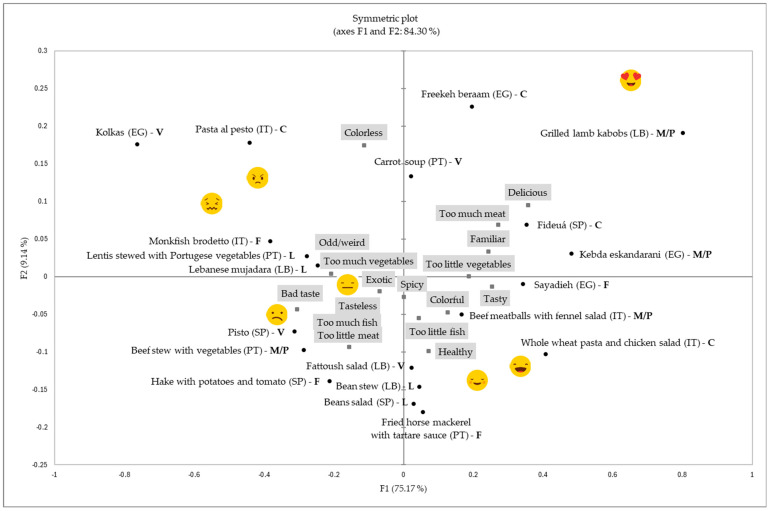
Symmetric plot showing the relationship among recipes, emojis, and labels for Spanish and Lebanese children. Legend: Labels have been highlighted in grey to differentiate them from recipes clearly. The origin of each recipe is provided, with country abbreviations in brackets (SP: Spain, IT: Italy, LB: Lebanon, PT: Portugal, and EG: Egypt). Below the name and origin of each recipe, the main ingredient is indicated in bold text (V: Vegetables, M/P: Meat/Poultry, F: Fish, L: Legumes, and C: Cereals).

Once the sorting task was fulfilled, Spanish and Lebanese children (*n* = 134) were asked to complete a CATA activity and label the different groups they had made using different kinds of descriptors. The results of this second task are shown in Figure 3, which shows the symmetric plot with the relationship among recipes, emojis, and labels. Approximately 84% of the variation in the model was explained by F1 and F2, with F1 being clearly linked to acceptance. In general, children seemed to link concepts such as “too much vegetables” and “bad taste” with the recipes grouped under the emojis 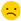 and 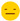, concepts such as “healthy”, “too little fish”, and “tasty” under the emojis 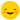 and 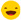, and concepts such as “too much meat”, “familiar”, and “delicious” with the recipes grouped under the 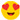 emoji. In addition, the concepts “too little vegetables” and “tasty” seemed to be linked to positive emojis. These responses suggested that children had a positive predisposition about healthy options, although a negative emotional response to “too much vegetables”.

## 4. Discussion

The present study illustrates the assessment of a set of 20 different recipes by Mediterranean children, using a sorting task that allowed classifying recipes by emojis and increasing understanding of their perception. In general, participants seemed to classify recipes according to their main ingredient, demonstrating a composition-based approach, similarly as previously reported by Riquelme et al. [23] when studying senior adults’ perception of different kinds or desserts. The results further confirmed that recipes classified under the most preferred categories predominantly contained meat and cereals, and the least accepted recipes were those in the vegetable-based category. These results were consistent with the ones recently reported by Piochi et al. [24], who studied meal perceptions in Italian primary school cafeterias. Pasta, meat, and fruits were the most liked foods, while fish, vegetables, and legumes were the least. The results from the present study showed some exceptions; for example, despite being categorized within the “cereals” group, Pasta al Pesto was not among the most favored recipes. The visual representation in the image may have influenced this perception. The green coloration of the pasta dish, which children might have associated with “vegetables”, could have significantly impacted their perception, given the documented and observed lower preference for the vegetable food category [25,26]. Walsh et al. [27] found children preferred red, green, orange, and yellow foods, suggesting a significant color effect. However, their study’s focus on candies may not generalize to broader food preferences, such as full meals.

Familiarity has been previously reported as a significant factor for food acceptability [28,29], although it does not always directly correlate with liking because of the influence of prior vital experiences [30]. The results of the present study suggested that Italian and Lebanese children were driven by familiarity, while Spanish showed a higher acceptance of foreign recipes. Proserpio et al. [31] reported minimal differences in neophobia scores among children aged 9–12 across Finland, Italy, Spain, Sweden, and the UK, but the results of the present study suggested that Italian children seemed to exhibit higher neophobia toward unfamiliar recipes than Spanish children. The ultimate causes producing food neophobia have not been fully recognized, although some factors, such as early exposure [32], parental influence [33], or even emotional stages [34], could have a considerable influence on children’s profile. Acceptability testing of millet-based recipes in Indian schools indicated that familiarity with the ingredients and traditional cooking methods contributed to acceptance [5]. A previous study has shown significant differences in the use of specific ingredients among Mediterranean cuisines [35], sometimes driven by sociocultural differences such as religion (e.g., pork and wine). Although the identification of ingredients most similar to their own culture in the photos could have driven children’s responses, the impact seemed to be different in Spain, Italy, and Lebanon, causing the groups of children to answer differently. Further studies are needed to better understand the origin of these differences in perceptions and preferences between countries; a greater understanding could be useful in developing targeted strategies to decrease food neophobia and increase consumption of healthy recipes and ingredients in children from different Mediterranean areas. Some of the strategies previously suggested to increase children’s acceptance of vegetables include involving them in cooking activities [36] or increasing the variety and vegetable options in the menu [37]. Because Lebanese children seemed to like recipes based on vegetables over children from other countries, further studies should aim to identify the determinants of these inter-group differences.

In addition to the sorting task, Spanish and Lebanese participants defined each recipe group through labeling, using descriptors such as “too much vegetables”, “bad taste”, and “odd/weird”. These labels were specifically associated with recipes grouped under emojis that expressed negative emotions. Recipes with vegetables and fish were the ones most frequently categorized in these groups, followed by recipes for which the main ingredients were legumes. These results confirmed different research showing that vegetables were among the least preferred and consumed food groups across different European cultures [25,29,38]. The observed preference for raw vegetables and salads over cooked vegetables, in alignment with previous research, may be attributed to the undesirable alterations in textural and visual properties that accompany the cooking of vegetables [26]. Although no tasting of the recipes was conducted, some associations were made by children, such as adding the label “bad taste” to vegetable-based recipes. Given that taste and flavor are primary determinants in children’s food selection [2], it seemed logical that less favored recipes were associated with “bad taste”. The recipes that received higher hedonic ratings, mainly composed of cereals and meat, were labeled as “tasty”, “familiar”, and “delicious” by Spanish and Lebanese children. Colorfulness and tastiness have been reported as positively associated with acceptance/preference and, as is known, the role of these sensory properties on food choice [2,39], but results of the present research seemed to suggest that these labels were “automatically” associated with these recipes because none of the recipes was currently tasted. In addition, some of the most disliked recipes, such as “pisto”, based on vegetables, could have been objectively labeled as “colorful” because of including many different ingredients with a variety of colors. The findings from the present research supported previous studies suggesting that foods with high caloric value are some of the most preferred [38], and positive labels were directly linked to these samples. Pasta, rice, and meat have ranked among the most popular foods among Spanish children, while fish has received lower hedonic ratings in previous studies [25,40,41], confirming the results of the present research.

The present study shows relevant data on children’s perception of recipes, a critical population in the process of establishing long-term dietary habits because evidence has shown that food habits formed in childhood persist into adulthood [40]. In addition to providing data about recipes’ acceptance, this study shows an example of a sorting task using dish photographs and confirms the utility of the proposed method to explore children’s reactions to different Mediterranean recipes. Varela & Salvador [21] proposed a structured sorting task where children had to sort twelve food items into four pre-determined groups. Children were able to group the products considering healthiness and their own hedonic response. During the present research, in addition to grouping by emoji (hedonic), the inclusion of descriptive labels added value by allowing a deeper understanding of the children’s responses. A key consideration in designing the data collection procedure was ensuring that the activity was child-friendly in terms of materials, content, and duration because the protocols must be adapted to the subject’s cognitive abilities [21,23]. Previous studies showed that children between 7 and 11 years old have developed independent product sorting and analysis skills [21,42]. In addition, the sorting task using images was proven as an effective tool for studying children’s multi-dimensional perceptions [21,40]. Based on the methodology suggested by Varela & Salvador [21], the present study effectively gathered information in a simplified manner while maintaining efficiency, combining sorting and liking assessments in a single activity with emojis.

## 5. Conclusions

The results of the present study suggested that Mediterranean children primarily classified recipes based on their main visual ingredients, employing a composition-based approach similar to that observed in older adults. Notable cross-cultural differences in recipe acceptance were found among Mediterranean children; while Italian and Lebanese children appeared to be more influenced by familiarity, Spanish ones showed a greater openness to foreign recipes. This study confirmed a general trend of lower preference for vegetable-based dishes across the sample, with negative labels like “too much vegetables” and “bad taste” frequently associated with this category. The visual sorting task combined with emoji ratings and labeling proved to be a valuable, child-friendly methodology for assessing initial visual perceptions of diverse Mediterranean recipes across cultures.

While the current study provides valuable insights into children’s initial perceptions of recipes based on visual cues and identifiable ingredients, several limitations should be addressed in future research. This study did not consider the potentially significant impact of the current flavor and texture of the dishes, which are directly affected by cooking methods. The lack of additional countries participating in the research (e.g., Portugal or Egypt), which could have provided broader insights, could also be considered a limitation of the study. Subsequent studies should expand upon these findings by including a greater variety of recipes, more countries or regions (e.g., Scandinavia vs. Mediterranean basin), different types of labels, and emoji-based group classifications. Further studies are needed to prove the robustness of the methodology and confirm the cross-cultural equivalence of emoji usage in expressing acceptance/feelings among children. Despite the potential influence of task speed and emoji familiarity on the results, the proposed activity proved valuable for exploring children’s perceptions within and across cultures; the methodology proved to be a useful tool for investigating children’s perceptions within a region and across cultures. It is also important to note that specific ingredients, such as pork, were deliberately omitted from the recipes to prevent biases related to religious considerations. Finally, studies involving children may face an additional limitation related to societal changes over time; findings from previous studies conducted a few years ago might not fully generalize to contemporary children because of evolving social norms or technological advancements. Therefore, the interpretation of the findings of the present study would benefit from more recent investigations about children’s food and meal choices and perceptions.

## Figures and Tables

**Table 1:** List of recipes used during the sorting task conducted by children from the Spanish, Lebanese, and Italian schools.

Recipe	Origin	Main Ingredient	Visual Ingredients
Monkfish Brodetto	Italy	Fish	Fish, onion, tomato puree, carrot, olives, capers, raisins, parsley
Pasta al pesto	Italy	Cereals	Pasta, pesto sauce, burrata, basil
Carrot soup	Portugal	Vegetables	Carrot cream, pepper, sprouts
Kolkas	Egypt	Vegetables	Taro root, chard, rice
Freekeh beraam	Egypt	Cereals	Pasta (freekeh), onion, chicken, spring onion
Hake with potatoes and tomato	Spain	Fish	Fish, potatoes, tomatoes, sweet corn
Lentils stewed with Portuguese vegetables	Portugal	Legumes	Lentils, cabbage, pumpkin, soy sprouts, carrot, onion, zucchini, chives
Sayadieh	Egypt	Fish	Fish, rice, onions, parsley
Lebanese mujadara	Lebanon	Legumes	Lentils, rice, onion, yogurt
Grilled lamb kabobs	Lebanon	Meat/poultry	Meat, green and yellow bell peppers, onion, parsley
Beans salad	Spain	Legumes	Beans, lettuce, tomato, olives, pickled cucumber, onion
Beef meatballs with fennel salad	Italy	Meat/poultry	Meatballs, fennel, capers, parmesan cheese
Lebanese bean stew	Lebanon	Legumes	Beans, chicken, tomato paste, tomatoes, cilantro
Fideuá	Spain	Cereals	Pasta, prawns, cuttlefish, lemon, tomato
Kebda eskandarani	Egypt	Meat/poultry	Beef, tomato, green peppers, bread, mint
Fattoush salad	Lebanon	Vegetables	Lettuce, cherry tomato, croutons, cucumber, green pepper, radish, green onion, sumac
Whole wheat pasta and chicken salad	Italy	Cereals	Pasta, chicken, Cherri tomato, zucchini, olives, basil
Beef stew with vegetables	Portugal	Meat/poultry	Beef, carrot, potatoes, green beans, peas, parsley
Pisto	Spain	Vegetables	Peppers, zucchini, onion, tomato sauce, chives
Fried horse mackerel with tartare sauce	Portugal	Fish	Fish, tartare sauce (mayonnaise, onion, flour, capers, chives, etc.), lemon

**Table 2:** ANOVA results of recipe assessments by factor. Different letters indicate groups that were significantly different (Tukey HSD).

Country	Acceptance (Mean)
Lebanon	4.1 a
Spain	4.1 a
Italy	3.7 b
*p*-value	<0.0001
Main ingredient	Acceptance (mean)
Meat/poultry	4.5 a
Cereals	4.2 b
Fish	3.9 c
Legumes	3.6 d
Vegetables	3.6 d
*p*-value	<0.0001
Recipe Origin	Acceptance (mean)
Lebanese	4.1 a
Egyptian	4.1 a
Italian	4.0 ab
Portuguese	3.9 ab
Spanish	3.8 c
*p*-value	0.007

## Data Availability

The original contributions presented in the study are included in the article, further inquiries can be directed to the corresponding author.
